# Editorial: Plant sensing and computing - PlantComp 2022

**DOI:** 10.3389/fpls.2024.1384726

**Published:** 2024-02-27

**Authors:** Michiel Stock, Tom De Swaef, Francis wyffels

**Affiliations:** ^1^ KERMIT, Department of Data Analysis and Mathematical Modelling, Faculty of Bioscience Engineering, Ghent University, Ghent, Belgium; ^2^ Institute for Agricultural, Fisheries and Food Research (ILVO), Merelbeke, Belgium; ^3^ Imec, Ghent University, Ghent, Belgium; ^4^ IDLAB-AIRO - Ghent University, Ghent, Belgium

**Keywords:** modeling, sensors, phenotyping, hyperspectral imaging, machine learning, precision agriculture

How can computing and sensing technology contribute to fundamental and applied research in plant sciences? This was the central theme of the second edition of the PlantComp Workshop on Plant Sensing and Computing^1^, organized in October 2022 in Ghent. This topic can be examined in two ways: *for* plants and *by* plants (**Figure 1**).

**Figure 1:**
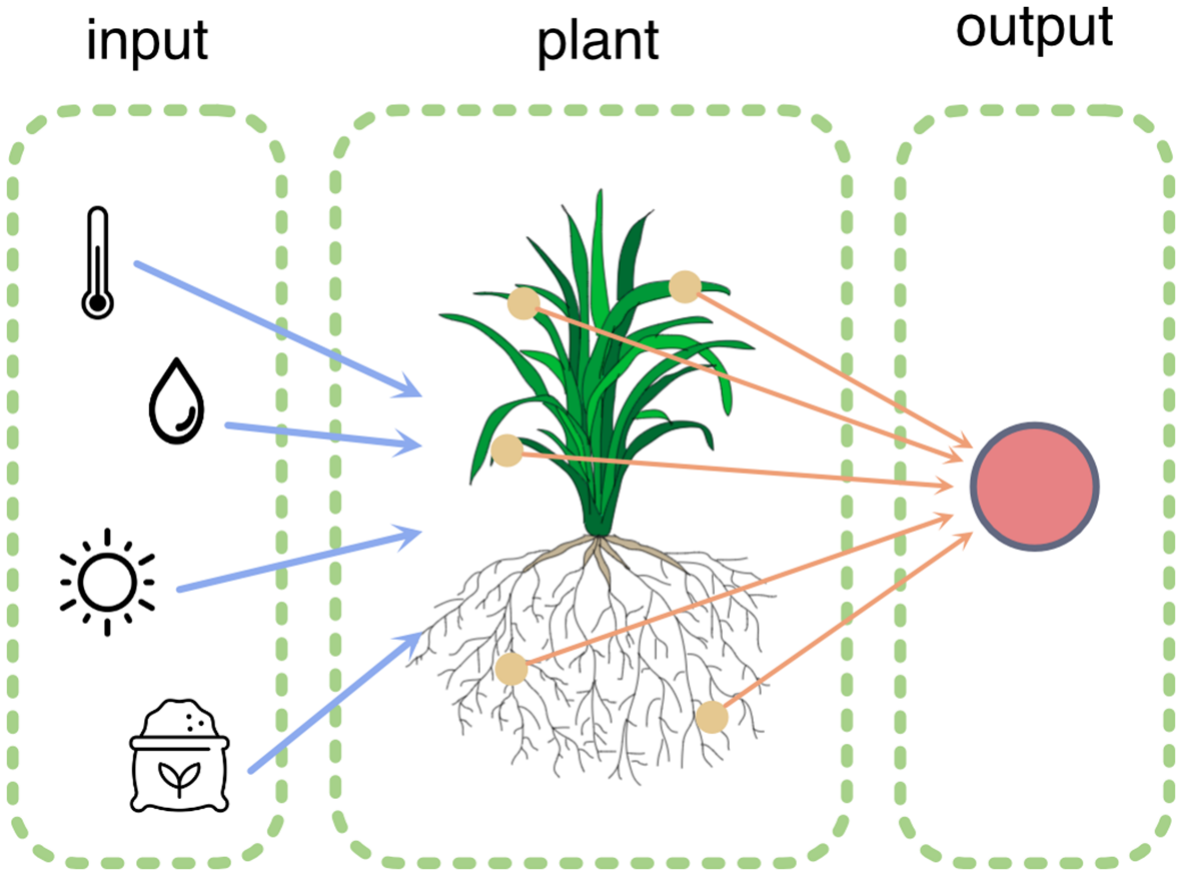
The plant actively senses its environment (blue arrows) and processes this information by modifying its morphology and physiology. These processes can be measured via various sensors (orange arrows).

As crop scientists, ecophysiologists, and plant breeders, we want to use sensors to understand how plants interact with their environment, whether we can monitor their health status, and quantify their phenotype. New sensing technologies such as hyperspectral imaging or impedance spectroscopy expand our horizons of new plant processes we can gain insight into. These technologies yield large quantities of data, for which we have powerful machine learning tools at our disposal. However, such data is of limited value if they cannot integrate into existing mechanistic knowledge or serve as a sensible data-to-decision pipeline for smart agriculture. Digital agriculture, applying big data and precision technology in agriculture (Ingram et al., 2022), will undoubtedly play a pivotal role in sustainable global food production.

On the other hand, plants are active organisms that perform various sensing activities - many alien to us mammals - to understand their environment. Plants process these collected inputs to compute an appropriate response by, e.g., modifying their growth or releasing volatile organic compounds. Given the radically different life cycle and architecture of plants compared to animals (for example, their lack of a central nervous system), plant’s mechanisms for information processing are radically different. The modular build of plants seems to lend itself to some form of swarm intelligence similar to social insects (Baluška et al., 2010). Various conceptual and experimental papers have shown the computational capacities of plants (Pieters et al., 2022; Trewavas, 2014; van Schijndel et al., 2022). A deeper insight into how plants perform cognition is obviously valuable scientifically, there is also great potential for applied plant scientists. If one can understand plants as agents with their own goals and programming, one can hack this to influence their growth, change their morphology, and improve, e.g., crop yield.

Our Research Topic includes seven papers that explore the themes of plant sensing and computation. We can broadly distinguish three major topics:

1. Spectral identification of plant health;
2. Alternative sensing methods;
3. Models for decision support in crop science.

## Spectral identification of plant health

Multi- and hyperspectral imaging enables the assessment of plant traits by capturing information across a broad spectrum of wavelengths in two spatial dimensions and has demonstrated its value across various settings such as laboratories, greenhouses, or fields (Sarić et al., 2022). The work “*Spectral characterization and severity assessment of rice blast disease using univariate and multivariate models*” by Mandal et al. showed the potential of satellite hyperspectral imaging to detect rice blast. Nonlinear machine learning methods, such as support vector regression, provide a reliable way to estimate the disease severity based on the near-infrared spectrum. Similarly, “*Ultra-High-Resolution UAV-Imaging and Supervised Deep Learning for Accurate Detection of Alternaria solani in Potato Fields*” by Wieme et al. used a convolutional neural network (CNN) to robustly detect potato blight. To this end, they collected a valuable dataset of high-resolution RGB UAV images of symptomatic and non-symptomatic potato crops during various years and disease stages. Finally, “*Modeling the spatial-spectral characteristics of plants for nutrient status identification using hyperspectral data and deep learning methods*” by Okyere et al. used a hybrid CNN that exploits both spectral and spatial information of the plants to predict phosphor and nitrogen nutritional status of cowpeas and quinoa plants.

## Alternative sensing methods

Many alternative non-invasive approaches exist for plant monitoring and phenotyping. In “*Plant impedance spectroscopy: a review of modeling approaches and applications*,” Van Haeverbeke et al. review how impedance spectroscopy, a method originally devised for studying electrical fuel cells, is applied to monitor plants’ ecophysiology and crop quality. The work “*Fast screening of total nutrient contents in strawberry leaves and spent growing media using NIRS*” by Vandecasteele and Van Waes showed the potential of near-infrared reflectance spectroscopy to characterize the nutrient content in strawberry leaves. Though their method does require the destruction of samples, it can accurately quantify the concentration of nutrients such as N, P, K, Ca, and Mg in leaves.

## Models for decision support in crop science

The last two papers relate to decision-support tools for optimal crop growth. “*Usefulness of cultivar-level calibration of AquaCrop for vegetables depends on the crop and data availability*” by Coudron et al. explore calibration of the AquaCrop model for high-value crops. A successful quantification of uncertainty of such decision support systems is vital to deduce an optimal irrigation regime. Finally, the article “*Plant Science in the Age of Simulation Intelligence*” by Stock et al. reviews the latest developments in computational intelligence relating to applied plant science. They investigate nine simulation intelligence motifs for transformative potential in plant and crop sciences.

## Author contributions

MS: Writing – original draft. TS: Writing – review & editing. FW: Writing – review & editing.

## Footnotes

1
https://plantcomp.ugent.be/
